# Drug Resistance in Hematological Malignancies

**DOI:** 10.3390/ijms21176091

**Published:** 2020-08-24

**Authors:** Patrick Auberger, Jerome Tamburini-Bonnefoy, Alexandre Puissant

**Affiliations:** 1 Team “Myeloid Malignancies and Multiple Myeloma”, Université Cote d’Azur, Inserm U1065/C3M, 06204 Nice, France; 2 Cochin Hospital, Assistance Publique–Hôpitaux de Paris, 75014 Paris, France; jerome.tamburini@aphp.fr; 3 INSERM U944, Hôpital St Louis, 75010 Paris, France; alexandre.puissant@inserm.fr

Hematological malignancies define a highly heterogeneous set of blood-, bone marrow-, and organ-associated diseases with highly variable prognoses that constantly relapse upon treatment. They account for 1.2 million new cases each year worldwide and represent around 7% of all newly diagnosed cancers. Among them, leukemia represents a group of hematological cancers that arise in blood-forming cells in the bone marrow and lead to an accumulation of abnormal blood cells in the BM (bone marrow) and the bloodstream. Worldwide, more than 400,000 people are diagnosed with leukemia each year, accounting for 2.5% of all diagnosed cancers. Each year, an estimated 100,000 new patients suffering leukemia are diagnosed in Europe and around 60,000 are diagnosed in the USA. While all age groups can be affected, leukemia is the most common pediatric tumor. On the other hand, lymphoma arises in immune cells called lymphocytes (T or B lymphocytes) in the lymph nodes, spleen, thymus, and bone marrow, but also in other organs of the body. Worldwide, over 500,000 people are diagnosed with lymphoma, with non-Hodgkin lymphoma representing around 85% of the total cases. Each year, an estimated 120,000 new lymphoma patients are diagnosed in Europe and around 80,000 are diagnosed in the USA.

Nowadays, leukemia and lymphoma can be treated by a plethora of drugs or drug combinations, including chemotherapy, targeted therapies, immunotherapies, immune checkpoint inhibitors, and CAR-T cells (chimeric antigen receptor-T cells). These therapies have significantly improved the management of patients, even leading to cures in some cases. Although chronic myelogenous leukemia and promyelocytic leukemia represent archetypes of the beneficial impact of targeted therapies, most other forms of leukemia and lymphoma remain a major public health concern. Resistance to all types of currently available therapies is a general hallmark and major drawback of leukemia and lymphoma, and significantly accounts for relapse and failure of treatments. Indeed, whatever the kind of treatment, malignant hematopoietic cells consistently develop cellular strategies to adapt to and survive from therapeutic drugs. Such adaptations may involve different molecular and cellular mechanisms, including the acquisition of mutations. In addition, the modulation of the signaling pathways involved in the regulation of apoptosis, autophagy, proteostasis, proliferation, differentiation, metabolism, epigenetic modifications, and oncogenes or tumor suppressors represent additional processes that may lead to therapy-induced resistance. Other potential mechanisms of resistance arise from the tumor stromal niche, for instance, through cytokine and growth factor production or exosome secretion. 

This special Issue of *IJMS* encompass twelve articles dedicated to providing an update of mechanisms of resistance to therapies (from conventional treatments to targeted and immunotherapies) in the course of treatment of hematological malignancies [1,2,3,4,5,6,7,8,9,10,11,12]. It also covers some of the mechanisms by which alterations of biochemical or signaling pathways can be therapeutically exploited to improve therapies. The hematological malignancies tackled in this issue essentially range from myelodysplastic syndromes and acute myeloid leukemia to acute T cell leukemia and lymphoma. 
